# Pharmacokinetics of Meloxicam in Different Animal Species: A Comprehensive Review

**DOI:** 10.3390/vetsci11110519

**Published:** 2024-10-24

**Authors:** Raul de la Puente, Raquel Diez, M. Jose Diez, Nelida Fernandez, Ana M. Sahagun, Jose M. Rodriguez, Juan J. Garcia, Cristina Lopez

**Affiliations:** Pharmacology, Department of Biomedical Sciences, Veterinary Faculty, Institute of Biomedicine (IBIOMED), University of Leon, 24071 Leon, Spain; rpueg@unileon.es (R.d.l.P.); mjdiel@unileon.es (M.J.D.); mnferm@unileon.es (N.F.); amsahp@unileon.es (A.M.S.); jmrodl@unileon.es (J.M.R.); jjgarv@unileon.es (J.J.G.); clopcd@unileon.es (C.L.)

**Keywords:** pharmacokinetics, animal species, meloxicam, absorption, distribution, metabolism, excretion

## Abstract

Meloxicam is a non-steroidal anti-inflammatory in the oxicam group. It has been extensively used in human and veterinary medicine for their anti-inflammatory, analgesic and antipyretic activities. Meloxicam has shown high therapeutic potential for disorders such as osteoarthritis, musculoskeletal disorder, acute respiratory infection, puerperal septicemia, mastitis and mastitis–metritis–agalactia syndrome. Although meloxicam pharmacokinetic has been described for numerous species, no paper summarizes the existing literature on this field. Thus, the aim of this review was to carry out a review of the literature on the pharmacokinetics of meloxicam in different animal species and gather the data in a single review article. A comprehensive review of the available literature in the PubMed, Web of Science and Scopus databases was performed. Meloxicam shows good bioavailability after oral and parenteral administration in most animal species (85–95%), with the lowest values in sheep after oral administration. It presents a rapid distribution with a small volume of distribution, which can be attributed to relatively high ionization state of meloxicam at physiological pH and its high plasma protein binding (close to 99%). It is extensively metabolized in the liver in several inactive polar metabolites, which are excreted, like unchanged meloxicam in urine and feces. Meloxicam also shows a long elimination half-life and low clearance.

## Simple Summary

Meloxicam has been extensively used in human beings and animals for ameliorating pain and inflammation associated with a wide range of disorders such as osteoarthritis, acute respiratory infection or mastitis, due to its anti-inflammatory, analgesic and antipyretic activities. Although meloxicam pharmacokinetics has been described for numerous species including dogs, cats, goats, sheep, horses, cattle, pigs and other species of veterinary interest, no paper summarizes the existing literature on this field. This article reviews the pharmacokinetics of meloxicam in several animal species. Meloxicam kinetics shows good bioavailability after oral and parenteral administration and a long elimination half-life. It is extensively metabolized in the liver into inactive metabolites. These and traces of unchanged meloxicam are excreted in urine and feces. Important changes in pharmacokinetic parameters have been observed among the species studied, as well as in the dosage required to achieve clinically effective plasma concentrations. Meloxicam kinetics vary according to the route of administration, formulation, animal species, age, body condition and physiological status, all of which may contribute to differences in drug efficacy. Drug pharmacokinetics are closely related to their pharmacological efficacy, so it is important to know their behavior in each species in which is used.

## 1. Introduction

Meloxicam (4-hydroxy-2-methyl-N-(5-methyl-2-thiazolyl)-2H-1,2-benzothiazine-3-carboxamide-1,1-dioxide) is a non-steroidal anti-inflammatory (NSAID) of the oxicam class group that preferentially inhibits cyclooxigenase-2 isoform (COX-2). Therefore, the drug blocks the conversion of arachidonic acid into prostaglandins (PG), such as PGE_2_, that induce inflammation, pain and fever [1,2,3]. Meloxicam has been extensively used in human and veterinary medicine due to its anti-inflammatory, analgesic and antipyretic activities. In veterinary medicine, this drug is effective in the treatment of osteoarthritis, musculoskeletal disorders, acute respiratory infection (as pneumonia), puerperal septicemia, mastitis and mastitis–metritis–agalactia syndrome [4,5,6,7,8]. Furthermore, its use is recommended in painful and inflammatory processes, such as castration or dehorning in several animal species [9,10,11].

Meloxicam kinetics shows good bioavailability after oral and parenteral administration, long elimination half-life (t_1/2_) and low gastrointestinal toxicity, making it an ideal and suitable NSAID to be used in animals [12]. It is extensively metabolized in the liver into inactive metabolites mainly through the cytochrome P450 2C pathway. Metabolites and traces of unchanged meloxicam are excreted in urine and feces.

Although meloxicam pharmacokinetics has been described for numerous species including goats, sheep, horses, cattle, dogs, cats, pigs and other species of veterinary interest, no paper summarizes the existing literature on this field. There is lot of interspecies variation in the pharmacokinetic profile of meloxicam and hence its disposition kinetics cannot be extrapolated from one species to another.

In this study, a comprehensive literature review was carried out to provide an overview of the pharmacokinetics of meloxicam in several animal species.

## 2. Materials and Methods

Literature data collection is essential for review papers because its quantity and quality directly determine the effectiveness of article visualization.

In the present review, a literature search was performed in three electronic databases, PubMed, Web of Science and Scopus, with no lower limit of publication date, in order to find the studies within the scope of our subject. The published literature until 1 June 2024 was searched.

To ensure the comprehensiveness of the literature search, the following search terms were used: “pharmacokinetic*”, “meloxicam”, “sheep”, “goat*”, “cattle”, “pig*”, “horse*”, “llama*”, “camel*”, “yak”, “reideer*”, “swine”, “cow*”, “dog*”, “cat*”, “absorption”, “distribution”, “metabolism”, “excretion”, using as Boolean operators AND and OR. Multiple search strings were set, as: TS = ((“pharmacokinetic” AND “meloxicam”) AND (“sheep” OR “goat*” OR “cattle” OR “pig*” OR “horse*” OR “llama*” OR “camel*” OR “yak” OR “reideer*” OR “swine” OR “cow*” OR “dog*” OR “cat*”)); TS = (“pharmacokinetic” AND “meloxicam” AND “animal species”).

The preliminary search yielded 1475 records. The selection criteria were then applied (full-text access, written in English and presence of pharmacokinetic parameters) and after removing duplicates and irrelevant content, 1298 papers were rejected. Papers selected (177) were evaluated by reading the titles and abstracts, and 109 articles were included in this phase. Then, the full text was reviewed and finally 74 articles were selected.

The document type included research article and review, and only peer-reviewed papers were included. Also, bibliographies of review articles were regularly screened for potentially relevant articles and pharmacokinetic case reports that did not appear in the initial search.

## 3. Results and Discussion

Numerous pharmacokinetic studies of meloxicam in different animal species have been carried out including sheep [4,5,13,14,15,16,17,18,19,20], goats [6,9,10,15,21,22,23,24,25], yak [26], cattle [11,27,28,29,30,31,32,33,34,35,36,37,38,39,40,41,42], buffaloes [19,43,44], llama [45], camels [46], reindeers [47], horses [48,49,50,51,52,53,54,55,56,57,58], pigs [7,59,60,61,62,63,64,65,66], dogs [67,68,69,70,71,72,73,74,75,76,77,78] and cats [79,80]. Meloxicam can be administered by oral, intravenous, intramuscular or subcutaneous routes, depending on the species. The doses studied varied between 0.2 and 2 mg/kg, being the dosage recommended by the manufacturers in these species between 0.4 and 0.6 mg/kg.

Despite its potent analgesic effect, the approved therapeutic use of meloxicam is limited to several species, including cattle, pigs, horses, dogs and cats. However, meloxicam is not currently approved by the European Medicines Agency (EMA) and Food and Drug Administration (FDA) to be used in small ruminants, such as sheep or goats. On the other hand, it has recently been approved by the Therapeutic Goods Administration (TGA) in Australia [8,21].

Pharmacokinetic parameters of meloxicam after different routes of administration (oral, intravenous, intramuscular and subcutaneous administration) in the different animal species are summarized in Table 1, Table 2, Table 3, Table 4, Table 5, Table 6 and Table 7. Some of the pharmacokinetic parameters reported in several studies were recalculated or transformed into the same units to facilitate the comparison of their values.

### 3.1. Sheep

Several authors [4,5,13,14,15,16,17,18,19,20] evaluated the pharmacokinetics of meloxicam after oral, intravenous, intramuscular and subcutaneous administration in sheep (Table 1).

All studies included in this review described the pharmacokinetics of meloxicam in this animal species with a non-compartmental method after oral [13,16], intramuscular [5] or subcutaneous administration [4,5,14,17,18]. Regarding intravenous administration, we found two studies in which the authors reported that meloxicam kinetics was best fitted by a two-compartmental model [15,19], but others like Stock et al. [16] and Gungor et al. [20] indicated that it was best described following a non-compartmental model.

**Table 1:** Pharmacokinetic parameters of meloxicam in sheep ^a^.

Ref	Route	Gender	Pk	Dose	F	C_max_	t_max_	AUC_0–∞_	V_d_	MRT	t_1/2_	Cl
		and N	Model	(mg/kg)	(%)	(μg/mL)	(h)	(μg·h/mL)	(L/kg)	(h)	(h)	(mL/kg·h)
[13]	PO	3♀, 3 Ct	NC	1	-	1.94	8.00	38.15	0.36	17.20	9.49	26.20
[16]	PO	1♀, 5 Ct	NC	1	71.00	1.78	24.00	74.30	0.28	31.50	15.50	13.20
[13]	PO	3♀, 3 Ct	NC	1 *	-	1.65	4.00	23.90	0.55	15.20	9.20	41.80
[15]	IV	6♀	TC	0.5	-	-	-	31.88	0.25	15.13	10.85	16.00
[16]	IV	5 Ct	NC	0.5	-	-	-	55.23	0.19	19.10	15.30	9.00
[19]	IV	6♀	TC	0.5	-	-	-	9.55	-	9.62	6.50	52.20
[20]	IV	6♀	NC	0.5	-	-	-	26.66	0.25	13.17	10.33	18.76
[20]	IV	6♀	NC	1	-	-	-	85.16	0.30	26.08	19.75	11.74
[20]	IV	6♀	NC	2	-	-	-	347.78	0.19	33.46	24.74	5.75
[5]	IM	6♀	NC	1	-	9.77	2.08	174.70	0.11	17.36	12.63	6.00
[5]	IM	6♀	NC	2	-	15.30	2.83	320.90	0.11	18.10	12.79	6.00
[4]	SC	5–6♀	NC	0.5	-	1.53	4.33	23.77	-	16.85	8.93	-
[18]	SC	6♀	NC	0.5	-	1.53	4.33	23.70	-	16.90	-	-
[5]	SC	6♀	NC	1	-	6.96	4.67	129.89	0.12	16.36	10.82	8.00
[17]	SC	6♀	NC	0.5 **	-	1.06	4.00	49.38	-	41.50	12.10	-
[17]	SC ^†^	6♀	NC	0.5	-	3.24	6.70	84.10	-	20.50	15.20	-
[5]	SC	6♀	NC	2	-	15.94	5.00	376.91	0.11	21.61	14.28	5.00
[14]	SC ^†^	6♀	NC	2	-	1.58	10.00	76.19	1.25	40.83	31.40	30.00

Ref = reference; Route: route of administration; N = number of animals; Pk = pharmacokinetic; ^a^ = adult animals; ♀ = female animals; Ct = castrated male; F = bioavailability; C_max_ = maximum plasma concentration; t_max_ = time to reach maximum plasma concentration; AUC_0–∞_ = area under the plasma concentration–time curve from time 0 to infinity; V_d_ = volume of distribution; MRT = mean residence time; t_1/2_ = elimination half-life; Cl = clearance; PO = oral; IV = intravenous; IM = intramuscular; SC = subcutaneous; ^†^ = use of SRM (sustained-release meloxicam) * = daily dose during 10 days; ** = daily dose during 3 days; NC = non-compartmental; TC = two-compartmental

#### 3.1.1. Absorption

Only one study evaluated the bioavailability of meloxicam after its oral administration at a single dose of 1 mg/kg in sheep [16], showing high bioavailability, close to 71%. Single oral dose data have limited clinical application, as many conditions requiring treatment with anti-inflammatory analgesics do not resolve within a single day, and multi-day therapy is often warranted and prescribed. In this sense, Depenbrock et al. (2021) [13] studied the pharmacokinetics of meloxicam bottle after a single oral dose and after a daily dose during 10 days. These authors determined values of C_max_ (maximum plasma concentration) (1.94 µg/mL) after single oral dose similar to those previously reported (1.78 µg/mL) [16]; however, the value of t_max_ (time to reach maximum plasma concentration) was lower (8 h) than that reported by Stock et al. (24 h) [16]. In the same line, Dunbar et al. (2019) [17] studied the pharmacokinetics of meloxicam after single subcutaneous doses of SRM and after daily dose of conventional formulation during 3 days. Values of C_max_ (3.24 µg/mL) and t_max_ (6.70 h) after a single dose of SRM were higher than those reported after single dose (C_max_ = 1.53 µg/mL; t_max_ = 4.33 h) [4,18] and daily dose during 3 days of conventional formulation of meloxicam (C_max_ = 1.06 µg/mL; t_max_ = 4.00 h) [17].

Woodland et al. (2019) [5] indicated that meloxicam after intramuscular and subcutaneous administration has a high bioavailability similar to those determined after oral meloxicam administration [16]. In the study developed by Woodland et al. (2019) [5], in which the plasma pharmacokinetic prolife of meloxicam after intramuscular and subcutaneous administration to sheep was evaluated, C_max_ after a dose of 2 mg/kg was similar after intramuscular (15.30 µg/mL) and subcutaneous administration (15.94 µg/mL), being lower when using a dose of 1 mg/kg (C_max_ = 9.77 after IM; C_max_ = 6.96 after SC). Moreover, t_max_ values were higher when using subcutaneous administration, approximately at 5 h, than after intramuscular administration (approximately at 2 to 3 h). After subcutaneous administration, different authors studied the pharmacokinetics of a sustained release meloxicam formulation (SRM). The C_max_ of the SRM (1.58 μg/mL [14]; 3.24 μg/mL [17]) was lower than that reported for a conventional formulation (15.94 μg/mL) [5]. T_max_ ranged between 6.7 and 10 h for the SRM, being higher than the value reported in the conventional formulation, 4.67–5 h [5].

The AUC_0–∞_ (total area under the plasma concentration versus time curve from time 0 to infinity) after oral administration of 1 mg/kg ranged between 23.90 μg·h/mL and 74.30 μg·h/mL [13]. After intravenous administration of 0.5 mg/kg, AUC_0–∞_ was between 9.55 μg·h/mL [19], 26.66 μg·h/mL [20], 31.88 μg·h/mL [15] and 55.23 μg·h/mL [16], obtaining higher values by increasing the dose (85.16 μg·h/mL after 1 mg/kg and 347.78 μg·h/mL after 2 mg/kg) [20]. After intramuscular and subcutaneous administration of the same doses (0.5) [4,5,14,17], the reported AUC_0–∞_ values were similar to the intravenous route.

In the study developed by Stock et al. [16], the delayed mean absorption time (12.40 h) suggested that meloxicam absorption in sheep occurred mostly distal to the four-compartment gastric tract, with primarily intestinal absorption; it also suggested drug storage within the rumen. This finding contrasts with meloxicam absorption in monogastric animals such as horses, in which absorption is relatively rapid, occurring over a long section of the gastrointestinal tract [12,16,49].

#### 3.1.2. Distribution and Metabolism

In 2007, Shukla et al. [15] determined the pharmacokinetic of meloxicam in sheep and goats following intravenous administration of 0.5 mg/kg. This study showed that meloxicam was rapidly distributed with half-lives for distribution of 0.42 h, similar to that obtained in goats (0.37 h) [22] and horses (0.40 h) [56]. The volume of distribution (V_d_) obtained after 0.5 mg/kg ranged between 0.19 L/kg and 0.25 L/kg [15,16,20]. Similar values were reported after intravenous administration of higher doses of 1 and 2 mg/kg (0.30 L/kg and 0.19 L/kg) [20].

The volume of distribution (V_d_) obtained in sheep after oral administration ranged between 0.19 L/kg and 0.25 L/kg [15]. The small V_d_ obtained in these studies indicates that the drug distributes primarily in the extracellular space.

The volume of distribution reported by several authors after oral [13,16], intramuscular [5] and subcutaneous [4,5,17] administration ranged between 0.11 L/kg and 0.55 L/kg. The small volume of distribution of meloxicam may be attributed to the relatively high ionization state of meloxicam at physiological pH and its high plasma protein binding. Meloxicam, similar to most NSAIDs, is highly protein-bound (greater than 99%) and can be distributed into the extracellular space as well as penetrate other tissues.

Regarding mean residence time (MRT), Depenbrock et al. [13] reported values of 17.20 h after oral administration of meloxicam. However, studies conducted by Stock et al. [16] showed higher MRT values after an oral dose of 1 mg/kg. On the other hand, values indicated by Depenbrock et al. [13] are in accordance with those reported after intravenous [15,16,19,20] and intramuscular administration [5]. After subcutaneous administration of meloxicam in sheep, several studies reported values between 16.36 and 21.61 h [5], while other studies indicated higher values (approximately 40 h) [14,17].

Meloxicam is extensively metabolized in the liver into three inactive polar metabolites: 5′-carboxy metabolite, 5′-hydroxymethyl metabolite and the metabolite formed by the cleavage of the side chain [15].

#### 3.1.3. Excretion

Depenbrock et al. [13] determined values of t_1/2_ (9.49 h) after single oral dose of 1 mg/kg, similar to that reported previously by Stock et al. [16]. The t_1/2_ reported after subcutaneous administration ranged between 8.93 and 15.20 h (dose of 0.5–2 mg/kg) [4,5,17], 31.40 h (dose of 2 mg/kg) [14], and around 12 h after intramuscular administration [5]. The authors indicated that these differences may be due to differences in the formulation and/or individual animal differences. In sheep, after intravenous administration (0.5 mg/kg), the values of t_1/2_ obtained by different authors (6.50–10.85 h) [15,16,19,20] were similar to those obtained in goats for the same dose (6.73–12.8 h) [10,15,21,22,23].

Regarding clearance (Cl), the mean values obtained by authors after oral administration are different: 13.20 mL/kg·h [16] to 26.20 mL/kg·h [13]. The same happens after intravenous administration with values ranging from 9 mL/kg·h [16] to 16 mL/kg·h [15] or even higher, 52.20 mL/kg·h [19]. After subcutaneous and intramuscular administration, Cl was very similar and next to 6 mL/kg·h [5], being this value lower than that obtained after oral administration.

### 3.2. Goats

The pharmacokinetics of meloxicam have been studied after oral, intravenous, intramuscular and subcutaneous administration in goats by different authors [6,9,10,15,21,22,23,24,25], using doses between 0.35 and 1 mg/kg (Table 2). It is worth mention that pharmacokinetic studies carried out in lactating and nonlactating goats [21,23], revealed that the lactating condition had no significant effects on pharmacokinetics of meloxicam after intravenous and intramuscular administration.

After intravenous administration of meloxicam at the dose rate of 0.5–1 mg/kg, pharmacokinetic parameters of meloxicam were calculated by non-compartmental methods according to the majority of studies included in this review. The same happens for the oral, intramuscular and subcutaneous routes; however, Wani et al. [25], Shukla et al. [15] and de Vito et al. [23] reported that meloxicam kinetics was best described by a two-compartmental open model when administered orally (0.35 mg/kg) and intravenously (0.5 and 1 mg/kg). In the same way, De Vito et al. reported that meloxicam kinetics was best described by a one-compartmental model after intramuscular administration of 0.5 mg/kg [23].

**Table 2:** Pharmacokinetic parameters of meloxicam in goats.

Ref	Route	Gender	Pk	Dose	F	C_max_	t_max_	AUC_0–∞_	V_d_	MRT	t_1/2_	Cl
		and N	Model	(mg/kg)	(%)	(μg/mL)	(h)	(μg·h/mL)	(L/kg)	(h)	(h)	(mL/kg·h)
[25] ^b^	PO	5♂	TC	0.35	80.50	1.61	8.28	52.29	0.11	24.54	11.55	6.00
[22] ^e^	PO	5♀	NC	0.5	96.49	0.71	14.33	24.21	-	24.33	10.69	24.21
[10] ^b^	PO	8♀	NC	0.5	79.00	0.73	15.00	23.24	-	25.40	11.80	-
[24] ^d^	PO	6 Ct	NC	0.5	-	0.70	6.00	17.61	0.44	-	10.70	29.10
[21] ^b^	IV	6♀	NC	0.5	-	-	-	27.50	0.34	11.90	12.80	10.00
[21] ^a,b^	IV	6♀	NC	0.5	-	-	-	26.60	0.32	13.70	13.00	10.00
[22] ^e^	IV	5♀	NC	0.5	-	-	-	25.09	0.37	14.43	12.73	20.31
[10] ^b^	IV	8♀	NC	0.5	-	-	-	29.73	0.25	13.90	10.90	17.90
[15] ^b^	IV	5♀	TC	0.5	-	-	-	19.23	0.25	9.37	6.73	30.00
[23] ^b^	IV	6♀	TC	0.5	-	-	-	26.50	0.26	13.88	9.96	19.38
[9] ^c^	IV	6♂	NC	1	-	-	-	41.10	0.45	18.26	13.50	24.43
[6] ^b^	IV	5♂	TC	1	-	-	-	43.92	0.28	10.22	8.10	22.56
[21] ^b^	IM	6♀	NC	0.5	103.70	1.70	2.37	-	0.39	13.90	13.40	20.00
[21] ^a,b^	IM	6♀	NC	0.5	75.30	1.47	2.91	-	0.43	14.50	11.20	20.00
[23] ^a,b^	IM	6♀	OC	0.5	105.93	1.41	3.73	28.07	0.28	-	10.82	18.77
[22] ^e^	SC	5♀	NC	0.5	98.24	1.91	3.20	24.65	-	15.67	15.16	-

Ref = reference; Route: route of administration; N = number of animals; Pk = pharmacokinetic; ♀ = female animals; ♂ = male animals; Ct = castrated male; F = bioavailability; C_max_ = maximum plasma concentration; t_max_ = time to reach maximum plasma concentration; AUC_0–∞_ = area under the plasma concentration–time curve from time 0 to infinity; V_d_ = volume of distribution; MRT = mean residence time; t_1/2_ = elimination half-life; Cl = clearance; PO = oral; IV = intravenous; IM = intramuscular; SC = subcutaneous; ^a^ = lactation; ^b^ = adult animals; ^c^ = kids (4–5 months old); ^d^ = kids (5–8 months old); ^e^ = kids (12–14 months old); NC = non-compartmental; OC = one-compartmental; TC = two-compartmental.

#### 3.2.1. Absorption

Bioavailability reported by Karademir et al. [22] was similar following oral and subcutaneous administration (96.49% and 98.24%, respectively), but the value after oral treatment was higher than that reported previously by Ingvast-Larsson et al. (79%) [10] and Wani et al. (80.5%) [25]. In addition, intramuscular bioavailability is almost complete, ranging between 75.30 and 105.93% [21,23].

Studies in goats observed values of mean absorption half-life of 2.84 h, indicating a good meloxicam absorption rate after oral administration [25]. After intramuscular administration, the half-life of the absorption phase was around 1.08 h [23].

Values of C_max_ reported by several authors after oral administration of 0.5 mg/kg ranged between 0.70 and 0.73 μg/mL and were lower than those observed after intramuscular (1.41–1.70 μg/mL) [21,23] and subcutaneous (1.91 μg/mL) [22] administration of the same dose. With regard to t_max_, values were higher after oral administration (6–15 h) [10,22,24] regarding the intramuscular and subcutaneous routes (2.37–3.73 h and 3.20 h, respectively. In 2014, Wani et al. [25] determined de pharmacokinetics of meloxicam following oral administration of 0.35 mg/kg. The time to achieve maximum plasma concentration (C_max_ = 1.61 μg/mL) was 8.28 h.

The AUC_0–∞_ in goats is lower than reported in sheep [16] and cattle [31] after intravenous administration. For this route, the values of AUC_0–∞_ for lactating (26.60 μg·h/mL) and nonlactating goats (27.50 μg·h/mL) determined by Kim et al. (2019) [21] were similar to those reported previously: 26.50 μg·h/mL [23]; 25.09 μg·h/mL [22] and 29.73 μg·h/mL [10]. In contrast, higher values were demonstrated by Wani et al. [6] (43.92 μg·h/mL) and Tekeli et al. [9] (41.10 μg·h/mL), and lower values by Shukla et al. [15] (19.23 μg·h/mL).

#### 3.2.2. Distribution and Metabolism

Following intravenous administration, meloxicam was rapidly distributed in goats, with disposition half-life (t_1/2α_) values between 0.37 and 0.53 h [6,15,23]. These values are in line with those reported in sheep (0.42 h) [16] and horses (0.40 h) [56]. The short half-life value indicates that meloxicam is distributed quite rapidly in this animal species.

The mean volume of distribution of meloxicam reported in 2020 by Tekeli et al. in goats after intravenous administration was 0.45 L/kg [9], which was larger than previously reported for goats (0.25–0.37 L/kg) [10,15,21,22,23], sheep (0.17 L/kg) [16], calves (0.17 L/kg [38]; 0.19 L/kg [31]) or llamas (0.24 L/kg) [45]. Values obtained by Tekeli et al. after intravenous administration were similar to those reported after oral and intramuscular administration [21,23,24]. As already mentioned above, the small volume of distribution obtained in the different studies indicates that the drug distributes primarily in the extracellular space of sheep and goats.

Regarding MRT, studies conducted in goats showed slight differences between oral, intravenous, intramuscular and subcutaneous administration of 0.5 mg/kg of meloxicam. Values after intravenous (9.37–18.26 h) [6,9,10,15,21,22,23], intramuscular (13.9–14.5 h) [21] and subcutaneous (15.67) [22] administration were lower than those reported by oral administration (24.33–25.40 h) [10,22,25]. These values were lower than those indicated in sheep [4,5,13,16,17] and cattle [29,30,31,37,39,42]; what it reflects is that meloxicam is eliminated at a faster rate in goats than in sheep and cattle.

Meloxicam is primarily metabolized into inactive polar metabolites in the liver, and goats are known to have higher activities of the drug-metabolizing biotransformation enzyme cytochrome P450 2C9 [42]. This may explain the higher clearance measured in the different studies reported.

#### 3.2.3. Excretion

In this animal species, the t_1/2_ values obtained by the different authors were very similar using oral, intravenous, intramuscular and subcutaneous administration. The data obtained were close to 12 h [9,10,21,22,23,24,25]. After oral administration of 0.5 mg/kg, values were similar to previous reported in sheep [15]. Nevertheless, the value reported by Karademir et al. [22] after subcutaneous administration was slightly higher (15.16 h) than the previous data and other authors reported lower values after intravenous administration [6,15].

After oral administration of 0.5 mg/kg meloxicam, Cl in goats ranged between 24.21 and 29.10 mL/kg·h [22,24], being less than after intramuscular administration (18.77 mL/kg·h) [23]. Tekeli et al. [9] reported a Cl of 24.43 mL/kg·h after intravenous administration, which was in agreement with data previously reported in goats [10,22,23]. This value was higher than that reported in sheep [16] or llamas [45] and lower than that indicated in horses [51]. The authors suggest that the reason for the difference could be due to differences in animal breeds but also in the drug analysis technique.

### 3.3. Cattle

Pharmacokinetic studies have been carried out in cattle after oral, intravenous and subcutaneous administration of meloxicam, using doses between 0.2 and 1 mg/kg [11,27,28,29,30,31,33,34,35,36,37,38,39,41,42]. A summary of pharmacokinetic parameters can be seen in Table 3.

In cattle, after oral administration of different doses of meloxicam (0.5 and 1 mg/kg), studies included in this review indicated that they used non-compartmental methods [27,28,30,33,34,35,36,37,38,39,42]. The non-compartmental analysis is also used to describe the kinetic of meloxicam after subcutaneous administration of 0.5 mg/kg [11,28,30]. After intravenous administration, some authors [31] suggest that a two-compartmental open model is the best fit for pharmacokinetics of meloxicam, but others [27,38] performed a non-compartmental study.

**Table 3:** Pharmacokinetic parameters of meloxicam in cattle.

Ref	Route	Gender	Pk	Dose	F	C_max_	t_max_	AUC_0–∞_	V_d_	MRT	t_1/2_	Cl
		and N	Model	(mg/kg)	(%)	(μg/mL)	(h)	(μg·h/mL)	(L/kg)	(h)	(h)	(mL/kg·h)
[39] ^c^	PO	8♂	NC	0.5 *	-	4.52	88.50	239.32	0.20	48.82	25.66	5.72
[41] ^c^	PO	6♂	-	0.5	-	4.70	89.60	276.78	-	-	22.49	-
[35] ^d^	PO	6♂	NC	0.5	-	2.11	11.67	-	0.16	-	20.47	6.00
[42] ^c^	PO	6 -	NC	0.5	-	1.95	17.30	86.70	0.28	50.30	29.90	6.45
[42] ^c^	PO	6 -	OC	0.5	-	-	-	86.20	0.18	-	-	6.44
[42] ^e^	PO	6 -	NC	0.5	-	2.20	17.00	151.00	0.21	63.50	40.00	5.28
[42] ^e^	PO	6 -	OC	0.5	-	-	-	149.00	0.23	-	-	5.34
[27] ^a,f^	PO	6♀	NC	1	87.20	1.45	10.48	36.01	0.39	20.38	9.55	30.00
[27] ^b,f^	PO	6♀	NC	1	101.6	2.61	16.75	82.82	0.22	27.62	12.28	10.00
[28] ^b,f^	PO	6♀	NC	1	-	1.95	11.70	115.60	-	-	28.50	-
[38] ^c^	PO	6♂	NC	1	-	3.10	11.64	164.46	0.24	44.90	27.54	6.40
[33] ^e^	PO	10♂	NC	1	-	3.61	13.95	216.12	0.27	-	38.62	6.00
[34] ^f^	PO	6♀	NC	1	-	2.89	11.33	89.99	-	19.46	14.58	9.95
[36] ^a,f^	PO	10♀	NC	1	-	1.82	11.60	51.92	-	22.30	11.93	13.90
[36] ^b,f^	PO	10♀	NC	1	-	2.92	17.60	109.34	-	30.29	13.01	6.82
[37] ^d^	PO	7♂	NC	1	-	1.90	24.00	78.00	0.34	34.20	16.70	-
[30] ^d^	PO	12♂	NC	1	-	2.33	24.00	95.16	0.24	34.10	15.60	11.11
[29] ^f^	PO	5♀	NC	30	-	91.06	19.71	3301.30	0.18	33.27	13.79	9.09
[27] ^a,f^	IV	5♀	NC	0.2	-	1.22	0.13	8.26	0.29	11.30	8.23	30.00
[27] ^b,f^	IV	7♀	NC	0.2	-	1.06	0.23	16.30	0.31	24.16	17.31	10.00
[38] ^c^	IV	6♂	NC	0.5	-	-	-	82.34	0.17	28.19	20.35	6.10
[31] ^c^	IV	6♂	TC	0.5	-	-	-	81.02	0.19	31.24	-	6.64
[28] ^b,f^	SC	6♀	NC	0.5	-	1.59	5.33	39.60	-	-	12.50	-
[11] ^f^	SC	8♂	NC	0.5	-	1.96	2.83	50.29	0.24	21.30	16.10	9.94
[30] ^d^	SC	11♂	NC	0.5	-	2.37	3.70	64.45	0.18	22.60	16.20	7.98

Ref = reference; Route: route of administration; N = number of animals; Pk = pharmacokinetic; ♀ = female animals; ♂ = male animals; F = bioavailability; C_max_ = maximum plasma concentration; t_max_ = time to reach maximum plasma concentration; AUC_0–∞_ = area under the plasma concentration–time curve from time 0 to infinity; V_d_ = volume of distribution; MRT = mean residence time; t_1/2_ = elimination half-life; Cl = clearance; PO = oral; IV = intravenous; SC = subcutaneous; ^a^ = mid-lactation; ^b^ = post-partum; ^c^ = 4–6 months old; ^d^ = 6–8 months old; ^e^ = pre-ruminant (6–8 weeks old); ^f^ = adult animals; * = daily dose during 3 days; NC = non-compartmental; OC = one-compartmental; TC = two-compartmental.

#### 3.3.1. Absorption

Bioavailability determined in cattle after oral administration (87.20–101.60%) was consistent with the reviewed literature, showing that meloxicam is extensively absorbed after oral dosage [27].

The studies carried out to evaluate the maximum plasma concentration and the time to reach this maximum plasma concentration of meloxicam after the different routes of administration showed a C_max_ between 1.59 μg/mL (t_max_ = 5.33 h) and 2.37 μg/mL (t_max_ = 3.70 h) after subcutaneous administration and C_max_ between 1.45 μg/mL (t_max_ = 10.48 h) and 4.70 μg/mL (t_max_ = 89.6 h) after oral administration [11,27,28,30,33,34,35,36,37,38,39,41,42].

The AUC_0–∞_ was greater in cattle and calves receiving oral compared to subcutaneous meloxicam administration [27,28,29,33,34,35,36,37,38,39,41,42]. Values were around 39.60–64.45 μg·h/mL for the subcutaneous route [11,28,30]. The AUC_0–∞_ is an indicator of the total drug exposure and it is dependent on dose and rate of elimination. After oral administration, studies indicated that AUC_0–∞_ in calves [30,33,37,38,41,42] was greater than the AUC_0–∞_ reported in sheep (23.90–74.30 μg·h/mL with the dose of 1 mg/kg) [13,16] and goats (from 17.61 μg·h/mL at dose of 0.5 mg/kg to 52.19 μg·h/mL with 0.35 mg/kg) [10,22,24,25], indicating that meloxicam is better absorbed in calves than in small ruminant species. The values of AUC_0–∞_ reported by authors after intravenous administration in cattle ranged from 8.26 μg·h/mL to 16.30 μg·h/mL [27], observing higher values in the studies carried out in calves (81.02 μg·h/mL [31]; 82.34 μg·h/mL [38]). The dose used, the age and the different physiological conditions of the animals, together with interspecies variability, may have influenced these wide ranges in values.

#### 3.3.2. Distribution and Metabolism

The disposition half-life (t_1/2α_) of meloxicam after intravenous administration was 0.24 h, a value similar to that reported in yaks (0.21 h), thus suggesting rapid distribution of meloxicam [26]. Compared to these values, longer disposition half-life (t_1/2α_) of 0.42 h [15], 0.37 h [15] and 0.70 h [59], respectively, in sheep, goats and piglets have been reported, which suggest that distribution of meloxicam was faster in cattle compared to the other studies species and these differences may be attributed, according to the authors, to species-dependent anatomical, physiological and/or biochemical variations between different species [26].

Volumes of distribution (V_d_) were low irrespective the age of the animals, ranging between 0.17 and 0.31 L/kg after intravenous administration [27,31,38], and they were quite similar to those calculated for other routes of administration [11,27,29,30,33,35,37,38,39,42]. These low values indicated that meloxicam is mainly found in the vascular space as opposed to the extravascular space. Meloxicam is a drug that is highly bound to plasma proteins (96.5%), and its molecules are ionized at physiological pH in ruminants; therefore, it is mainly found in the vascular space [8,30,81]. The high protein binding also accounts for the low clearance values observed, not only in this animal species but also in others reported in this review.

Values reported for MRT after intravenous administration of 0.2 mg/kg ranged between 11.30 and 24.16 h [27], and after a dose of 0.5 mg/kg, between 11.30 and 31.24 h [31,38]. Values close to 21 h have been reported after subcutaneous administration [11,30], and after oral administration, studies indicated values between 19.46 and 44.90 h (dose of 1 mg/kg) [27,29,30,34,36,37,38] and 48.82 and 63.50 h (dose of 0.5 mg/kg) [39,42].

Meloxicam metabolites detected in cattle are pharmacologically inactive and include those found in other species (5′-hydroxymethyl, 5′-carboxy and oxalyl metabolites) as well as an additional polar metabolite [8]. Cytochrome P450 genes are highly polymorphic, and recent studies of select cytochrome P450 genes in cattle indicate differences in transcription between sexes and between the breeds studies [82,83].

#### 3.3.3. Excretion

In cattle, the t_1/2_ values obtained by Warner et al. (8.23 h) [27] were similar to those reported in yaks after intravenous administration [26], but were shorter compared to other studies carried out in cattle [27,38], sheep [15,16], goats [9,21,22] and buffalo (40.48 h) [44].

With regard to Cl, the mean values obtained by authors after oral, intravenous and subcutaneous administration are different: approximately 5.5 mL/kg·h [30,31,35,36,38,39,42] to 11 mL/kg·h [11,27,29,30,34,36] or even higher, e.g., 30 mL/kg·h [27]. After subcutaneous administration, calves had lower Cl than after oral administration, which is in agreement with the longer t_1/2_ of subcutaneous treatment than oral administration. The t_1/2_ (16.20 h) in calves was slightly higher than that reported for goats (15.10 h) [22] after subcutaneous meloxicam administration using the same dose of 0.5 mg/kg, while higher values of t_1/2_ (16.70 h [37]; 27.54 h [38]; 38.62 h [33]) have been reported following oral administration in calves.

Studies conducted by Warner et al. [27] to compare pharmacokinetics of meloxicam between healthy post-partum vs. mid-lactation dairy cattle showed values of Cl in post-partum of approximately half compared to mid-lactation cattle.

On the other hand, studies carried out by Fritz et al. [29] to evaluate the pharmacokinetics, milk residues and toxicity of a single oral high dose of meloxicam (30 mg/kg) in lactating dairy cattle indicated a t_1/2_ of 13.79 h; this value was similar to other data in cattle using a much smaller dose [27,30,34,36]. Oral clearance reported (9.09 mL/kg·h) was also similar to cattle studies using 1 mg/kg. These results suggest that excretion is not impacted by the increased dose.

Another study [34] determined the pharmacokinetic parameters of meloxicam in milk and plasma after oral administration and compared these parameters to those reported for ruminant beef calves in other studies [35,42]. The authors concluded that lactation did not appear to alter the plasma pharmacokinetic of meloxicam, just like in goats [21,23].

### 3.4. Buffalo, Llama, Camel, Yak and Reindeer

Few studies have been conducted on buffalo, llama, camel, yak or reindeer to determine the pharmaceutic parameters of meloxicam. Table 4 shows the values of the main pharmacokinetic parameters determined in the published studies reviewed after oral and intravenous administration [19,26,43,44,45,46,47].

In buffalo and yak, the pharmacokinetic profile of meloxicam after intravenous administration of 0.5 mg/kg was best fitted to a two-compartmental model [19,26,43,44]; however, studies carried out in llama and reindeer to determine the bioavailability and pharmacokinetics of meloxicam after oral and intravenous administration used non-compartmental analyses [45,47]. In Camels, the pharmacokinetics of meloxicam after intravenous administration (0.6 mg/kg) was also calculated by non-compartmental methods.

**Table 4:** Pharmacokinetic parameters of meloxicam in buffalo, llama ^a^, camel ^a^, yak ^a^ and reindeer ^a^.

Ref	Route	Gender	Pk	Dose	F	C_max_	t_max_	AUC_0–∞_	V_d_	MRT	t_1/2_	Cl
		and N	Model	(mg/kg)	(%)	(μg/mL)	(h)	(μg·h/mL)	(L/kg)	(h)	(h)	(mL/kg·h)
**Buffalo**												
[43] ^b^	IV	6♂	TC	0.5	-	-	-	118.81	0.14	32.89	21.51	4.38
[19] ^c^	IV	6♂	TC	0.5	-	-	-	55.54	-	23.51	40.48	10.20
[44] ^c^	IV	6♂	TC	0.5	-	2.13	0.083	-	0.21	23.51	40.48	10.20
**Llama**												
[45]	PO	6 -	NC	1	76.00	1.31	21.40	68.35	0.48	41.70	22.70	14.88
[45]	IV	6 -	NC	0.5	-	-	-	43.96	0.24	20.60	17.40	11.40
**Camel**												
[46]	IV	6♂	NC	0.6	-	-	-	346.70	0.09	53.70	40.20	1.94
**Yak**												
[26]	IV	5♂	TC	0.5	-	-	-	25.46	2.13	6.50	4.65	350
**Reindeer**												
[47]	PO	7♂	NC	0.5	49	1.82	6.00	-	-	27.80	19.30	-
[47]	IV	4♂	NC	0.5	-	-	-	-	0.13	22.00	15.20	3.98

Ref = reference; Route: route of administration; N = number of animals; Pk = pharmacokinetic; ^a^ = adult animals; ♂ = male animals; F = bioavailability; C_max_ = maximum plasma concentration; t_max_ = time to reach maximum plasma concentration; AUC_0–∞_ = area under the plasma concentration–time curve from time 0 to infinity; V_d_ = volume of distribution; MRT = mean residence time; t_1/2_ = elimination half-life; Cl = clearance; ^b^ = 3–4 months old; ^c^ = 6–7 months old; PO = oral; IV = intravenous; NC = non-compartmental; TC = two-compartmental.

#### 3.4.1. Absorption

Values of AUC_0–∞_ differ between studies carried out in buffalo calves. While Patel et al. [19] reported values after intravenous injection (0.5 mg/kg) of 55.54 μg·h/mL, Cagnardi et al. [43] indicated higher values (118.81 μg·h/mL).

In llamas, after oral administration of meloxicam, a C_max_ of 1.31 μg/mL occurred at approximately 21.40 h [45]. This route of administration demonstrated, in a pharmacokinetic study developed by Kreuder et al., a high degree of gastrointestinal absorption and oral bioavailability. After oral and intravenous administration of a single dose of 0.5 mg/kg, AUC_0–∞_ values were 68.35 μg·h/mL and 43.96 μg·h/mL, respectively. The AUC_0–∞_ in yak after intravenous administration of meloxicam was 25.46 μg·h/mL.

In reindeer [47], the bioavailability calculated was between 46 and 73%, and the C_max_ obtained after oral administration of 0.5 mg/kg was 1.82 μg/mL (t_max_ = 6.00 h).

#### 3.4.2. Distribution and Metabolism

The pharmacokinetic profile of meloxicam in buffalo was characterized by a fast disposition half-life (t_1/2α_) (0.09 h) and slow elimination half-life (21.51 h), and after a single intravenous injection of meloxicam at 0.5 mg/kg, this was quantifiable for several days with a mean concentration of 0.18 μg/mL at 96 h [43]. The V_d_ reported in buffalo calves was low, around 0.14 L/kg, in agreement with the data reported in cattle calves (0.19 L/kg) [31] after intravenous administration. As reported in several studies, the high protein binding of meloxicam (96.5–98%) [8] can justify the limited volume of distribution observed in buffalo calves. However, this high protein binding facilitates passage into areas of inflammation with the leakage of plasma proteins into exudate [45]. Regarding MRT, studies conducted in buffalo calves showed values of 32.89 h [43] and 23.51 h [19,44].

Studies in llamas showed a volume of distribution after oral administration of 0.48 L/kg and after intravenous administration of 0.24 L/kg [45]. MRT values ranged between 20.60 h to intravenous injection and 41.70 h to oral administration.

In camels, the plasma meloxicam prolife were characterized by fast distribution phase and a small V_d_ (0.09 L/kg) [46], much lower than that observed in the other animal species covered in this review.

In the study carried out by Ahmed et al. [26] in yak, thedisposition half-life (t_1/2α_) of meloxicam after intravenous administration in yak was 0.21 h, thus suggesting rapid distribution of meloxicam. Similar values were reported in cattle (0.24 h). Moreover, if we compare these values with those indicated in sheep, goats or piglets (0.37–0.70 h) [15,59], it can be seen that distribution of meloxicam was faster in yak and cattle. V_d_ was 2.13 L/kg; this value is in accordance with that reported by Ahmed et al. in cattle. However, it is higher than those reported by other authors, both in cattle and other animal species. The MRT of meloxicam was 6.50 h and was almost comparable with cattle in the study carried out by Ahmed et al. [26].

Meanwhile, V_d_ reported in reindeer after intravenous administration was 0.13 L/kg, and MRT 22.00 h (similar values are indicated for oral administration, 27.80 h) [47].

#### 3.4.3. Excretion

The t_1/2_ reported in buffalo calves was 21.51 h [43], being less than that indicated by other authors (40.48 h) [19,44]. The low Cl (4.38 mL/kg·h) is responsible for the rather long t_1/2_ and is influenced by the hepatic metabolism, which is necessary for meloxicam elimination from the body. Studies carried out by Patel et al. [19] in sheep and buffalo calves to compare pharmacokinetic parameters after intravenous administration indicated that the elimination kinetics of meloxicam differed significantly between both animal species, with relatively faster elimination in sheep.

The t_1/2_ following oral (22.70 h) versus intravenous (17.40 h) meloxicam administrations in llamas were similar to the results seen in other ruminant species studies [10,15,16,20,21,22,31,33,38,43].

In camels, a study developed by Wasfi et al. [46] showed a very slow Cl (1.94 mL/kg·h) and a very long t_1/2_ (40.20 h). The Cl indicated was lower than values reported in all other animal species.

Concerning yak, t_1/2_ was 4.65 h after intravenous administration [26]. This value contrasts with those obtained in reindeers (15.20 h) [47], llamas (17.40 h) [45], camels (40.20 h) [46] and buffalos (21.51 h [43]; 40.48 h [19,44]). In this sense, the Cl in this animal species was very high (350 mL/kg.h), showing that meloxicam elimination is much faster in yak than in the other ruminant species evaluated. Cl values were at the other extreme in reindeer (3.98 mL/kg.h) [47] and camel (1.94 mL/kg.h) [46]. This fact demonstrates that it is not always possible to extrapolate dosage from one species to another.

### 3.5. Horses

Several authors [48,49,50,51,52,53,55,56,57,58] compared the pharmacokinetic profile of meloxicam after oral and intravenous administration in horses, including in foals (Table 5).

In horses, after oral administration of 0.6 mg/kg, all studies included in this review indicated that the pharmacokinetic study was performed by non-compartmental methods [48,51,52,55,58]. However, after intravenous administration, some authors [50,56] suggest that a two- or three-compartmental open model is the best fit for pharmacokinetic of meloxicam, but others [48,49,51,53,57] suggest non-compartmental analysis.

Some studies [48,49] evaluated the influence of different formulations of meloxicam on the main pharmacokinetic parameters. Other investigations [50] focused on the possible influence of the state of health of the animals on the pharmacokinetic of meloxicam.

**Table 5:** Pharmacokinetic parameters of meloxicam in horses.

Ref	Route	Gender	Pk	Dose	F	C_max_	t_max_	AUC_0–∞_	V_d_	MRT	t_1/2_	Cl
		and N	Model	(mg/kg)	(%)	(μg/mL)	(h)	(μg·h/mL)	(L/kg)	(h)	(h)	(mL/kg·h)
[48] ^c^	PO ^a,1^	6♀, 1 Ct	NC	0.6	110.37	1.21	1.50	20.27	-	31.57	24.20	-
[48] ^c^	PO ^a,2^	6♀, 1 Ct	NC	0.6	88.27	2.08	1.00	17.89	-	14.58	13.17	-
[48] ^c^	PO ^a,3^	6♀, 1 Ct	NC	0.6	78.13	1.98	1.50	15.60	-	14.01	10.30	-
[48] ^c^	PO ^b,1^	6♀, 1 Ct	NC	0.6	96.55	0.85	1.00	20.60	-	47.55	34.08	-
[48] ^c^	PO ^b,2^	6♀, 1 Ct	NC	0.6	75.43	2.10	0.50	15.42	-	13.76	10.85	-
[48] ^c^	PO ^b,3^	6♀, 1 Ct	NC	0.6	90.11	2.70	0.75	18.26	-	14.82	12.33	-
[51] ^c^	PO ^a,2^	4♀, 4 Ct	NC	0.6	85.30	2.58	1.50	-	-	7.22	-	-
[51] ^c^	Poz ^b,2^	4♀, 4 Ct	NC	0.6	98.00	1.73	3.40	-	-	9.30	7.70	-
[58] ^d^	PO ^b,2^	7♀, 3♂	NC	0.6	-	0.92	2.62	11.28	-	-	10.24	-
[52] ^c^	PO ^a,2^	1♀, 7 Ct	NC	0.6	-	0.67	5.50	9.30	-	11.10	6.40	-
[52] ^c^	PO ^a,3^	1♀, 7 Ct	NC	0.6	-	0.71	2.50	8.41	-	9.60	6.50	-
[55] ^c^	PO ^b,3^	3♀, 1♂, 3 Ct	NC	0.6	-	1.58	3.48	11.22	0.41	7.20	5.25	55.33
[55] ^c^	PO ^b,3^	3♀, 1♂, 3 Ct	NC	0.6 *	-	2.07	1.24	14.19	0.32	-	4.99	44.27
[55] ^c^	PO ^3^	3♀, 1♂, 3 Ct	NC	0.6 **	-	1.81	1.93	11.73	0.37	7.18	4.73	54.24
[58] ^d^	PO	7♀, 3♂	NC	0.6	85–98	1.00	<1.5	-	-	-	2.48	154.00
[49] ^c^	IV	3♀, 3 Ct	-	0.25	-	-	-	-	0.24	3.48	6.17	72.00
[49] ^c^	IV	3♀, 3 Ct	-	0.5	-	-	-	-	0.23	2.74	5.29	86.00
[48] ^c^	IV	6♀, 1 Ct	NC	0.6	-	-	-	20.61	0.36	11.82	12.39	29.12
[50] ^c^	IV	3♀, 2♂, 3 Ct	NC	0.6	-	-	-	22.29	0.16	5.73	5.70	27.91
[50] ^c^	IV	3♀, 2♂, 3 Ct	TC/TR	0.6	-	-	-	22.00	0.19	6.62	6.88	28.14
[51] ^c^	IV ^a^	4♀, 4 Ct	NC	0.6	-	-	-	-	0.12	3.60	8.54	34.00
[53] ^c^	IV	3♀, 1♂, 1 Ct	NC	0.6	-	-	-	18.80	0.27	9.60	-	34.70
[57] ^c^	IV	6 -	NC	0.6	-	-	-	33.44	0.11	-	4.07	19.80
[56] ^c^	IV	5♀, 1 Ct	TC/TR	0.6	-	-	-	14.53	0.16	-	2.70	41.87
[49] ^c^	IV	3♀, 3 Ct	-	1.0	-	-	-	-	0.24	2.90	3.95	91.00
[49] ^c^	IV	3♀, 3 Ct	-	2.0	-	-	-	-	0.21	2.94	5.19	75.00

Ref = reference; Route: route of administration; N = number of animals; Pk = pharmacokinetic; ♀ = female animals; ♂ = male animals; Ct = castrated male; F = bioavailability; C_max_ = maximum plasma concentration; t_max_ = time to reach maximum plasma concentration; AUC_0–∞_ = area under the plasma concentration–time curve from time 0 to infinity; V_d_ = volume of distribution; MRT = mean residence time; t_1/2_ = elimination half-life; Cl = clearance; PO = oral; IV = intravenous; ^a^ = in fasted; ^b^ = fed; ^c^ = adult animals; ^d^ = foals; ^1^ = granule; ^2^ = suspension; ^3^ = tablets; NC = non-compartmental; TC = two-compartmental; TR = three-compartmental; * = daily dose for 7 days; ** = daily dose for 14 days.

#### 3.5.1. Absorption

Studies carried out by Mendoza et al. [48] in adult horses demonstrated values of bioavailability from 75.43% to 110.37% after oral administration (dose of 0.6 mg/kg) of different formulations (granule, suspension and tablets). The values obtained by Toutain et al. (85.30–98.00%) are also within this range [51]. In foals, studies demonstrated values of bioavailability from 85.00 to 98.00% [58].

Results yielded by Mendoza et al. [48] indicated faster t_max_ values in fed horses (t_max_ = 0.5 to 1 h) compared to fasted ones (t_max_ = 1 to 1.5 h), contrary to what was previously reported by Toutain et al. [51] (t_max_ fed horses = 3.40 h; t_max_ fasted horses = 1.50 h), after oral administration of meloxicam. The authors indicated that this discrepancy may be attributable to differences in experimental design. C_max_ ranged between 0.85 μg/mL and 2.70 μg/mL and AUC_0–∞_ between 15.42 μg·h/mL and 20.60 μg·h/mL after oral administration [48]. Values of C_max_ are in accordance with those indicated by other authors [48,51,52,55,58], observing lower values of AUC_0–∞_ in the pharmacokinetics studies carried out by Noble et al. [54] Vivancos et al. [52] and Vander Werf et al. [55].

The values of AUC_0–∞_ reported in horses after intravenous administration ranged from 18.80 μg·h/mL to 33.44 μg·h/mL [48,50,53,57]. Nevertheless, in ponies, lower values can be observed, 14.53 μg·h/mL [56].

Concerning foals, studies carried out by Radial et al. [58] indicated a peak plasma concentration of meloxicam approximating 1 μg/mL (t_max_ = above 1.5 h) after oral administration (0.6 mg/kg). Foals demonstrated rapid absorption of meloxicam after oral administration, which coincided with values reported in horses by different studies [48,55]; however, other studies carried out in horses by Toutain et al. [51], Noble et al. [58], Vivancos et al. [52] and Vander Werf et al. [55] indicated higher values of t_max_ (1.93–5.50 h).

#### 3.5.2. Distribution and Metabolism

Meloxicam protein binding indicated by Mendoza et al. [48] and Toutain et al. [49] was high, reaching a value close to 97.75–98.6%.

Toutain et al. [49] analyzed the pharmacokinetic of meloxicam using various doses (0.25; 0.5; 1 and 2 mg/kg). Authors indicated that they did not detect any significant effect of dose on any of the pharmacokinetic parameters, which suggests linearity in meloxicam’s disposition. So, V_d_ ranged from about 0.21 L/kg to 0.24 L/kg and MRT from 2.74 h to 3.48 h. Compared to other studies, several [50,51] indicated lower values of volume of distribution, while others [48,53] reported higher values, such as Mendoza et al. [48]. Regarding MRT and after intravenous administration, the values obtained by different authors [48,49,50,51,53] are lower than the values reported after oral administration of meloxicam—where some disparity can be observed—from 7.18 h to 47.55 h [48,51,52,55].

Meloxicam metabolites detected in horses are pharmacologically inactive and include those found in other animal species.

#### 3.5.3. Excretion

In the study carried out by Toutain et al. [49], after intravenous administration, the t_1/2_ and Cl were not influenced by dose, as happened with V_d_ and MRT. Elimination half-life after oral administration ranged from 2.70 h to 12.36 h [48,49,50,51,56,57]. It can be observed that the value reported by Mendoza et al. [48] was higher than those reported by other authors, while the lowest value corresponds to that reported in ponies [56]. Values of t_1/2_ after oral administration obtained from the reviewed studies ranged from 34.08 h to 4.73 h [48,51,52,55,58]. Foals showed a value of 2.48 h with a Cl of 154 mL/kg·h [58]. After intravenous administration, Toutain et al. reported high values of Cl around 80 mL/kg·h. Nevertheless, other authors indicated values around 20–40 mL/kg·h [48,50,51,53,56,57]. For the oral route, values indicated were similar to each other (44.27 mL/kg·h–54.24 mL/kg·h) [55].

### 3.6. Pigs

Several authors [7,59,60,61,62,63,64,65,66] compared the pharmacokinetic profile after oral, intravenous and intramuscular administration of meloxicam in sheep, using doses ranging between 0.4 and 2 mg/kg (Table 6).

Pairis-Garcia et al. [64], investigated the disposition of meloxicam in pigs after single-dose oral administration of 0.5 mg/kg body weight. These authors concluded that meloxicam kinetics was calculated by non-compartmental methods. This is also indicated by different authors [7,60,61,62,65] after intramuscular administration of meloxicam. Nevertheless, Fosse et al. [63] reported that meloxicam kinetics was best described by a one-compartmental model when administered intramuscularly at a dose of 0.6 mg/kg. Regarding the intravenous route, Pairis-Garcia et al. [64] indicated a non-compartmental analysis to describe the pharmacokinetics of meloxicam; nevertheless, Fosse et al. [59] indicated that profile meloxicam was adequately fitted by a two-compartmental open model.

**Table 6:** Pharmacokinetic parameters of meloxicam in pigs.

Ref	Route	Gender	Pk	Dose	F	C_max_	t_max_	AUC_0–∞_	V_d_	MRT	t_1/2_	Cl
		and N	Model	(mg/kg)	(%)	(μg/mL)	(h)	(μg·h/mL)	(L/kg)	(h)	(h)	(mL/kg·h)
[64] ^e^	PO	6♀	NC	0.5	87.00	1.07	2.40	11.61	0.43	9.01	6.83	43.08
[59] ^a^	IV	13 -	TC	0.4	-	3.28	-	8.03	0.19	3.50	2.70	61.00
[64] ^e^	IV	6♀	NC	0.5	-	5.71	-	13.27	0.16	4.26	6.15	37.80
[60] ^e^	IM	7 -	NC	0.4	-	1.20	0.37	4.65	0.41	4.15	3.34	90.00
[62] ^e^	IM ^1^	3♀, 3♂	NC	0.4	-	1.12	0.59	3.43	0.27	2.66	1.55	125.76
[62] ^e^	IM ^2^	3♀, 3♂	NC	0.4	155.98	0.82	2.33	5.35	0.38	6.16	3.74	85.54
[61] ^b^	IM	8 -	NC	0.4	-	1.79	0.28	17.28	0.25	8.75	6.56	24.90
[65] ^d^	IM	8♂	NC	0.4	-	1.58	1.21	10.75	0.24	5.80	4.46	40.00
[63] ^c^	IM	3♀, 9♂	OC	0.6	-	2.00	1.10	-	0.23	-	2.60	60.40
[7] ^e^	IM ^2^	6 -	NC	0.8	-	1.92	3.25	-	0.21	5.90	2.63	57.57
[7] ^e^	IM ^2^	6 -	NC	2	90.63	3.03	4.00	-	0.89	10.73	6.88	50.72
[66] ^b^	IM	12♂	-	1	-	3.12	0.50	17.13	0.18	-	3.94	20.00

Ref = reference; Route: route of administration; N = number of animals; Pk = pharmacokinetic; ♀ = female animals; ♂ = male animals; F = bioavailability; C_max_ = maximum plasma concentration; t_max_ = time to reach maximum plasma concentration; AUC_0–∞_ = area under the plasma concentration–time curve from time 0 to infinity; V_d_ = volume of distribution; MRT = mean residence time; t_1/2_ = elimination half-life; Cl = clearance; PO = oral; IV = intravenous; IM = intramuscular; ^a^ = 16–23 days old; ^b^ = 8 days old; ^c^ = 14 days old; ^d^ = 5 days old; ^e^ = adult animals; ^1^ = suspension; ^2^ = oil suspension; NC = non-compartmental; OC = one-compartmental; TC = two-compartmental.

#### 3.6.1. Absorption

In pigs, there was only one reference regarding the oral bioavailability of meloxicam [64]; Pairis-Garcia et al. demonstrated oral bioavailability of 87%, a value similar to that reported in the other animal species included in this review.

Pairis-Garcia et al. [64] determined the pharmacokinetic of meloxicam in mature swine after intravenous and oral administration and compared their results with those obtained in piglets by Fosse et al. [59]. C_max_ (5.71 μg/mL) in mature swine was greater than results reported in piglets (3.28 μg/mL) [59].

Following intramuscular administration of meloxicam, the peak plasma concentration was reached between 0.28 and 2.33 h [60,61,62,65] after 0.4 mg/kg; 1.10 h after 0.6 mg/kg [63]; 3.25 h after 0.8 mg/kg [7]; 0.5 h after 1 mg/kg [66]; and 4.00 h after 2 mg/kg [7]. In general, the values of C_max_ of meloxicam after intramuscular administration increased with dose. After 0.4 mg/kg, values of C_max_ ranged between 0.82 and 1.79 μg/mL [60,61,62,65], while at higher doses (0.6–2 mg/kg), it ranged between 1.92 and 3.12 μg/mL [7,63,66].

In mature swine, value of AUC_0–∞_ after intravenous injection (0.5 mg/kg) was 13.27 μg·h/mL; meanwhile, in piglets, values around 8.03 μg·h/mL were observed [59]. In the case of the intramuscular administration of the same dose (0.4 mg/kg), values reported in piglets (10–75–17.28 μg·h/mL) [61,65,66] were higher than those determined in adult pigs (3.43–5.35 μg·h/mL) [60,62]. On the other hand, after oral administration of meloxicam, AUC_0–∞_ and volume of distribution were 11.61 μg·h/mL and 0.43 L/kg, respectively [64].

#### 3.6.2. Distribution and Metabolism

In general, the volume of distribution is low for most NSAIDs in most animal species, and meloxicam is not an exception. Fosse et al. indicated a V_d_ of 0.19 L/kg [59] after intravenous administration of meloxicam in piglets. This value is in line with those reported by Pairis-Garcia et al. in mature swine (V_d_ = 0.16 L/kg) [64]. Plasma protein binding was not assessed in most of the reviewed studies, but in the study carried out by Busch et al. [12] in miniature pigs, plasma protein binding in vitro of meloxicam was already approximately 96%. This value is in accordance with values reported in other animal species. MRT indicated in mature swine (4.26 h) [64] was greater than results reported in piglets (3.50 h) [59] after intravenous administration.

Studies in piglets [61,63,65], showed that the volume of distribution was approximately 0.25 L/kg after intramuscular administration. This value is similar to those indicated by Li et al. [62] and Guo et al. [7] in adult pigs, but lower than those indicated by Nixon et al. [60] in adult pigs. Regarding MRT, the values obtained by the different authors [7,60,61,62,65] ranged from 2.66 h to 10.73 h after intramuscular administration; between 3.50 h and 4.26 h after intravenous injection [59,64] and 9.01 h after an oral dose of 0.5 mg/kg [64].

In pigs, less than 3% of the dose in excreted as unchanged meloxicam in urine [8]. Meloxicam has been shown to be extensively metabolized in miniature pigs, and the major metabolites found were the 5-hydroxymethyl and 5-carboxy metabolites, which are pharmacologically inactive [12].

#### 3.6.3. Excretion

The Cl reported by Fosee et al. [59] in piglets was 61 mL/kg·h after intravenous administration of meloxicam. This value is higher than that reported for mature swine (37.80 mL/kg·h) [64] and for other animal species like sheep [15,16,19], goats [6,9,10,15,21,22,23], cattle [27,31,38], horses [48,50,51,53,56,57] or buffalos [19,43,44]. The t_1/2_ indicated in piglets (2.70 h) [59] was lower than that observed in studies developed in mature swine (6.15 h) [64].

As for the intramuscular route, t_1/2_ ranged between 1.55 h and 6.88 h [7,60,61,63,65,66]. All studies included in this review showed that the t_1/2_ of meloxicam in pigs was shorter than other animals, exhibiting a rapid metabolism. Regarding Cl, studies carried out by Enouri et al. [61] indicated a value of 24.90. This value is lower than those reported by other authors (from 40.00 mL/kg·h to 125.76 mL/kg·h) [7,60,61,62,63,65], but higher than that reported by Viscardi [66] (20.00 mL/kg·h).

In 2021, Guo et al. [7] compared the pharmacokinetics of meloxicam oil suspension at different dosages (0.8 and 2 mg/kg) following intramuscular administration. In addition, they compared the results with the previous ones described by Li et al. [62] using a dose of 0.4 mg/kg (meloxicam oil suspension). MRT and t_1/2_ of 0.4 and 0.8 mg/kg did not show significant differences; however, both of these pharmacokinetic parameters at 2 mg/kg were markedly higher (MRT = 10.73 h; t_1/2_ = 6.88 h). Comparing these values with the conventional meloxicam injection [8,62], the MRT and t_1/2_ of meloxicam oil suspension were significantly prolonged at all dosages, indicating that the self-developed meloxicam oil suspension has a certain trend of sustained release.

Finally, after oral administration of 0.5 mg/kg meloxicam, t_1/2_ and Cl were 6.83 h and 43.08 mL/kg·h, respectively [64].

### 3.7. Dogs and Cats

Meloxicam pharmacokinetics have been studied in dogs after oral, intravenous and subcutaneous administration using doses between 0.2 and 0.8 mg/kg [12,67,68,69,70,71,72,73,74,75,76,77,78]. Yuan et al. [67] evaluated the pharmacokinetic profile of this drug following oral and transdermal administration. On the other hand, a study carried out by Less et al. [73] studied the bioequivalence of a meloxicam formulation administered as a transmucosal oral mist. Thus, Table 7 shows the summary of the pharmacokinetic parameters obtained.

Regarding the pharmacokinetic model, studies included in this review indicated that after oral administration of different doses of meloxicam (0.2–0.31 mg/kg), the pharmacokinetic parameters were established by a non-compartmental analysis [67,68,72,73,74]. After intravenous administration, some authors [12,75] also used non-compartmental methods to describe the pharmacokinetics of meloxicam, but Mahmood et al. [78] suggest that the model that best fits the data is the two-compartmental model.

Non-compartmental methods were also used to describe the kinetics of meloxicam after subcutaneous administration of 0.2 and 0.6 mg/kg [72], following transdermal administration of 1.25 mg/kg [67] and after its administration as a transmucosal oral mist [73].

In cats, the models that best fitted the pharmacokinetics of meloxicam after oral (0.1 mg/kg) [79] and subcutaneous administration (0.3 mg/kg) [80] were the one-compartmental and two-compartmental model, respectively.

**Table 7:** Pharmacokinetic parameters of meloxicam in Dogs ^b^ and Cats ^b^.

Ref	Route	Gender	Pk	Dose	F	C_max_	t_max_	AUC_0–∞_	V_d_	MRT	t_1/2_	Cl
		and N	Model	(mg/kg)	(%)	(μg/mL)	(h)	(μg·h/mL)	(L/kg)	(h)	(h)	(mL/kg·h)
**Dogs**												
[68]	PO	4♀, 4♂	NC	0.2	-	0.82	8.50	14.61	0.23	-	12.13	10.00
[77]	PO	4 -	-	0.2	90.24	-	-	24.29	-	-	-	-
[72]	PO	6♂	NC	0.2	-	0.29	4.00	-	-	-	-	-
[73]	PO ^3^	10♀	NC	0.2	-	0.61	6.50	27.66	-	-	30.00	-
[74]	PO ^a,3^	6♂	NC	0.21	-	0.82	3.33	29.42	0.31	31.34	30.48	7.41
[74]	PO ^a,4^	6♂	NC	0.21	-	0.83	2.08	28.16	0.31	31.14	28.77	7.74
[67]	PO ^1^	3♀, 3♂	NC	0.31	-	0.78	4.00	24.80	0.32	24.40	17.50	12.60
[70]	PO ^a,1^	9♂	-	0.75	-	3.19	7.89	128.62	-	-	27.67	-
[70]	PO ^a,1,A^	9♂	-	0.75	98.50	3.14	9.44	129.08	-	-	28.16	-
[70]	PO ^a,1,B^	9♂	-	0.75	100.52	3.08	8.44	128.95	-	-	30.86	-
[76]	PO ^a,5^	4♂	-	0.8	-	7.84	1.75	180.75	-	-	-	-
[76]	PO ^a,6^	4♂	-	0.8	-	6.23	5.50	142.53	-	-	-	-
[69]	PO ^1^	-	-	7.5 ^†^	-	0.32	7.90	-	-	-	33.50	-
[71]	PO ^1^	5 -	-	7.5 ^†^	-	3.18	8.00	-	-	-	-	-
[71]	PO ^2^	5 -	-	8.1 ^†^	-	4.55	1.38	-	-	-	-	-
[12]	IV	-	NC	0.2	-	0.46	-	21.50	0.32	34.80	24.00	10.00
[75]	IV	8♀	NC	0.2	-	-	-	23.00	0.26	28.10	17.21	10.50
[77]	IV	4 -	-	0.2	-	-	-	26.97	-	-	-	-
[78]	IV	8 -	TC	0.2	-	-	-	30.93	0.22	34.29	23.81	6.50
[72]	SC	5♂	NC	0.2	-	0.55	4.00	-	-	-	-	-
[72]	SC	5♂	NC	0.6	-	2.18	1.00	-	-	-	-	-
[67]	TD	3♀, 3♂	NC	1.25	-	0.025	11.30	1.19	-	36.10	36.60	-
[73]	TM	10♀	NC	0.2	-	0.62	4.48	26.82	-	-	29.60	-
**Cats**												
[79]	PO	3♀, 3♂	OC	0.1	-	-	-	-	0.25	-	25.70	6.56
[80]	SC	3♀, 3♂	TC	0.3	-	1.48	2.20	-	0.27	-	37.00	6.00

Ref = reference; Route: route of administration; N = number of animals; Pk = pharmacokinetic; ♀ = female animals; ♂ = male animals; F = bioavailability; C_max_ = maximum plasma concentration; t_max_ = time to reach maximum plasma concentration; AUC_0–∞_ = area under the plasma concentration–time curve from time 0 to infinity; V_d_ = volume of distribution; MRT = mean residence time; t_1/2_ = elimination half-life; Cl = clearance; PO = oral; IV = intravenous; SC = subcutaneous; TD = transdermal (gel); TM = transmucosal oral spray; ^1^ = tablets; ^2^ = SEDDS (powdered self-emulsified); ^3^ = suspension; ^4^ = ODF (oral disintegrating film); ^5^ = uncoated drug layered cores; ^6^ = enteric coated pellets; ^†^ = mg per animal; ^a^ = in fasted; ^b^ = adult animals; ^A^ and ^B^ = formulations under study; NC = non-compartmental; OC = one-compartmental; TC = two-compartmental.

#### 3.7.1. Absorption

Studies carried out by Mahmood and Ashraf [77] in healthy and clinically normal adult dogs demonstrated values of bioavailability of 90.24% after oral administration of 0.2 mg/kg. Slightly higher values were reported by Jin et al. [70] after an oral dose of 0.75 mg/kg (98.50 and 100.52%).

Several studies compared the bioavailability of meloxicam after oral administration of different pharmaceutical forms. One of these studies was carried out by Agarwal et al. [71], who compared the pharmacokinetic parameters of meloxicam administered as tablet (7.5 mg) vs. powdered self-emulsified lipid formulations (SEDDS) (8.1 mg). It was observed that oral bioavailability was greater for SEDDS than for tablets, which could be attributed to the differences in the dosage form due to an improvement in dissolution, as previously demonstrated in dissolution studies carried out by the same authors. In addition, the absorption of meloxicam from SEDDS was faster than meloxicam tablets, as observed by the shift in t_max_ reported in the study (1.38 min for SEDDS vs. 8.00 h for tablets) and in those carried out by other authors such as Tian et al. (7.90 h) [69] and Jin et al. (7.89–9.44 h) [70] using the same dose (0.75 mg/kg). This could be explained by the spontaneous formation of an emulsion upon drug release in the gastrointestinal tract that presents the drug in a solubilized form. Values of C_max_ indicated in these studies ranged between 3.08 μg/mL to 3.19 μg/mL after oral administration of tablets [70,71], being higher in the case of SEDDS (4.55 μg/mL) [71].

Values of C_max_ reported by several authors after oral administration of 0.2 mg/kg ranged between 0.29 and 0.83 μg/mL [67,68,72,73,74], and were similar than those observed after subcutaneous administration (0.55 μg/mL) [72] and after administered as transmucosal oral spray (0.62 μg/mL) [73]. Regarding to t_max_, values reported after oral administration ranged between 2.08 and 8.50 h [67,68,72,73,74]. Specifically, several authors [67,72] indicated a t_max_ of 4 h after an oral administration of 0.2 mg/kg, being this value equal to that reported for the subcutaneous route using the same dose [72]. After subcutaneous administration of higher doses (0.6 mg/kg), t_max_ was 1 h and C_max_ was 2.18 μg/mL [72].

The rate at which the drug reaches the systemic circulation is important for the drug used to treat acute conditions such as pain. After analyzing the studies that compared the bioequivalence of different formulations of meloxicam (tablets, SEDDS, suspension, oral disintegrating film, uncoated drug-layered cores and enteric coated pellets), we can conclude that after an oral dose of about 0.2 mg/kg, the fastest t_max_ was reached after administering oral disintegrating film (2.08 h) [74] compared with the t_max_ of tablets (4.00 h) [67] and suspension (3.30 h in fasted and 6.50 h eaten) [73,74]. Using higher oral doses, around 0.75 mg/kg, the fastest t_max_ was reached after SEDDS administration (1.38 h) [71], in comparison to the t_max_ of uncoated drug-layered cores (1.75 h) [76], enteric-coated pellets (5.50 h) [76], and tablets (values ranged from 7.89 h to 9.44 h) [69,70,71].

The AUC_0–∞_ after oral administration of 0.2 and 0.31 mg/kg ranged between 14.61 μg·h/mL and 29.42 μg·h/mL [67,68,72,74,75,76,78]. Similar values were observed after intravenous administration (AUC_0–∞_ was between 21.50 μg·h/mL and 30.93 μg·h/mL) [12,75,77,78] and when given as a transmucosal oral spray (26.82 μg·h/mL) [73]. Studies carried out with higher doses, around 0.75 mg/kg, showed AUC_0–∞_ values between 128.62 μg·h/mL and 180.75 μg·h/mL [70,76].

The AUC_0–∞_ (1.19 μg·h/mL) and C_max_ (0.025 μg/mL) obtained after transdermal administration [67] were much lower compared with those obtained after oral administration at different dosages (0.2 to 0.8 mg/kg) (AUC_0–∞_ = 14.61 to 180.75 μg·h/mL; C_max_ = 0.32 to 7.84 μg/mL) [68,69,70,71,72,73,74,76,77]. Although the systemic absorption was lower, the t_max_ after transdermal administration (11.30 h) was more prolonged than the oral administration (1.38–9.44 h, according to the different studies) [67,68,69,70,71,72,73,74,76].

On the other hand, in cats, after subcutaneous administration of meloxicam (0.3 mg/kg), a C_max_ of 1.48 μg/mL occurred at approximately 2.20 h [80], which is consistent with the data extracted from the technical sheets [84]

#### 3.7.2. Distribution and Metabolism

In dogs and cats, as in other animal species studied in this review, meloxicam is highly bound to plasma proteins, around 97% [12,75,79,80].

Studies in dogs [74] reported a volume of distribution of 0.31 L/kg after oral administration. This value is in agreement with that found previously by other authors [67,68,74]. After intravenous administration of the same dose (0.2 mg/kg), values were close to 0.26 L/kg [12,75,77,78].

Regarding MRT, studies conducted in dogs after oral and intravenous administration of 0.2 mg/kg showed similar results. Thus, the values ranged between 24.40 h and 31.14 h after oral administration [67,74] and between 28.10 h and 34.29 h for intravenous route [12,75,78]. MRT after transdermal administration was 36.10 h after a dose of 1.25 mg/kg [67].

Studies in cats showed a volume of distribution after oral administration of 0.25 L/kg [79] and after subcutaneous administration of 0.27 L/kg [80].

#### 3.7.3. Excretion

In this animal species, the t_1/2_ values obtained by the different authors were similar using oral and intravenous administration. After oral administration of 0.2 mg/kg, the values were around 28–30 h [73,74], although some studies carried out by Montoya et al. [68] and Yuan et al. [67] showed lower values, 12.13 h and 17.50 h, respectively. On the other hand, Jin et al. [70] and Tian et al. [69] reported values of t_1/2_ between 27.67 h and 33.50 h using a higher oral dose (0.75 mg/kg). After intravenous administration (dose of 0.2 mg/kg), values of t_1/2_ reported by different authors were 17.21 h [75], 24.00 h [12] and 23.81 h [78]. Finally, after transdermal administration of 1.25 mg/kg, t_1/2_ was 36.60 h [67], and administered as a spray into the mouth (transmucosal oral spray) using a dose of 0.2 mg/kg, it was 29.60 h [73].

Regarding Cl of meloxicam after oral and intravenous administration in dogs, this was approximately 10 mL/kg·h, with values ranging between 6.50 mL/kg·h and 12.60 mL/kg·h [12,67,68,74,75,78]. In cats, the reviewed studies reported a Cl of 6.56 mL/kg·h after oral administration (0.1 mg/kg) [79] and 6.00 mL/kg·h after subcutaneous administration of 0.3 mg/kg [80].

## 4. Conclusions

Important differences in meloxicam pharmacokinetics have been observed among the studies compared, even within the same animal species. These disparities may be attributed to the route of administration, dosage and formulation employed, the gender, age, body condition, and physiological status of the animals. All of them may contribute to differences in drug efficacy.

Generally, meloxicam shows good bioavailability after oral and parenteral administration in most animal species, with values around 85–100%, showing the lowest values in sheep after oral administration. Meloxicam presents a rapid distribution with a small volume of distribution, which can be attributed to its relatively high ionization state at physiological pH and its high plasma protein binding (close to 99%). It is extensively metabolized in the liver in several inactive polar metabolites, which are excreted, like unchanged meloxicam in urine and feces. Fecal excretion predominates in most of the species studied (like cattle, dogs and cats). Meloxicam also shows a long elimination half-life (ranging from 10–12 h in sheep, goats and horses to 24–36 h in dogs and cats) and low clearance (around 6–10 mL/kg·h to dogs and cats; about 10–30 mL/kg·h to sheep, goats and cattle; and, finally, about 30–90 mL/kg·h to horses and pigs), although differences have been observed between the reviewed studies. These differences may be due to differences in the meloxicam formulation used and/or individual animal differences. Finally, in general, the studies carried out in lactating and non-lactating animals revealed that the lactating condition had no significant effects on the pharmacokinetics of meloxicam.

## Data Availability

Not applicable.
